# Relationship between anal swab PCR for SARS-CoV-2 with gastrointestinal clinical manifestations and severity of COVID-19 infection in Indonesia

**DOI:** 10.12688/f1000research.128821.1

**Published:** 2023-03-31

**Authors:** Virly Nanda Muzellina, Murdani Abdullah, Juferdy Kurniawan, Aulia Rizka

**Affiliations:** 1 Division of Gastroenterology, Pancreatobiliary, and Digestive Endoscopy, Departement of Internal Medicine, Dr. Cipto Mangunkusumo National Central General Hospital, Faculty of Medicine Universitas Indonesia, Jakarta, Indonesia; 2 Division of Hepatobiliary, Department of Internal Medicine, Dr. Cipto Mangunkusumo National Central General Hospital, Faculty of Medicine Universitas Indonesia, Jakarta, Indonesia; 3 Division of Geriatric Medicine, Departement of Internal Medicine, Dr. Cipto Mangunkusumo National Central General Hospital, Faculty of Medicine Universitas Indonesia, Jakarta, Indonesia

**Keywords:** SARS-CoV-2, COVID-19, anal swab, gastrointestinal clinical manifestations, the severity of COVID-19

## Abstract

**Introduction**: Coronavirus disease 2019 (COVID-19) cases caused by severe acute respiratory syndrome coronavirus 2 (SARS-CoV-2) in Indonesia remain high. The virus can bind with ACE2 receptor which is not only found in the lungs, but also in the digestive tract. Thus, it allows SARS-CoV-2 infection in the gastrointestinal tract, gastrointestinal manifestations, and detection of viral RNA on anal swab using polymerase chain reaction (PCR). There hasn’t been similar study about the role of anal swab in Indonesia yet. Therefore, this study aims to determine the relationship between SARS-COV-2 anal swab PCR with gastrointestinal clinical manifestations, and the severity of COVID-19 in Indonesia.

**Methods**: This is an analytical study with cross-sectional design. Samples were obtained from hospitalized COVID-19 patients from July 2020 to January 2021. Demographic data, clinical manifestations, severity, and SARS-CoV-2 anal swabs PCR were collected using case report form.

**Results**: A total of136 patients were analyzed. 52 patients (38.2%) had positive SARS-CoV-2 anal swabs PCR and 84 patients (61.8%) had negative results. The most common gastrointestinal clinical manifestations were nausea and vomiting in 69 patients (50.7%), anorexia in 62 patients (45.6%), and abdominal pain in 31 patients (22.8%). There were 114 patients (83,8%) classified as mild-moderate symptoms and 22 patients (16,2%) classified as severe-critical symptoms. There was a statistically significant relationship between the gastrointestinal tract SARS-CoV-2 infection and gastrointestinal clinical manifestations (P=0.031). There was no statistically significant relationship between the gastrointestinal SARS-CoV-2 infection and the severity of COVID-19 infection (P = 0.844).

**Conclusions**: This study showed there is a significant relationship between SARS-CoV-2 anal swab PCR with gastrointestinal clinical manifestations. There is no significant relationship between anal swab PCR with the severity of COVID-19 infection. Further studies should aim to assess the relationship using larger samples while also assessing the possibility of fecal-oral transmission.

## Introduction

A virus with a new strain named coronavirus was discovered in the city of Wuhan, China on December 31 ^st^, 2019. On January 12, 2020, WHO (World Health Organization) announced this disease was named COVID-19 (coronavirus disease 2019) caused by severe acute respiratory syndrome coronavirus 2 (SARS-CoV-2). ^1^ Indonesia reported its first case in March, 2020. ^2^ The cases in Indonesia in March 2022 reached 5,974,646 with 154,062 cases of death and the Case Fatality Rate (CFR) of 2.58%. ^3^

SARS-CoV-2 can encode and express a spike glycoprotein (S) that can bind to the Angiotensin Converting Enzyme 2 (ACE2) receptor for entry into human cells. ^4^ The ACE2 receptors are not only found in the lungs, but also in the gastrointestinal tract which allows infection of enterocytes, causing gastrointestinal clinical manifestations, and detecting viral RNA on anal swab examination. ^4^ A systematic review found that 1 in 5 patients with confirmed COVID-19 had gastrointestinal symptoms. ^5^ Another study that evaluated gastrointestinal symptoms reported the prevalence of gastrointestinal symptoms including anorexia (26.8%), nausea and vomiting (10.2%), diarrhea (12.5%), abdominal pain (9.2%), and gastrointestinal symptoms. other (17.6%). ^6^^–^^8^

The Center of Disease Control and Prevention (CDC) has determined that the standard test for the diagnosis of COVID-19 is to use Reverse Transcription Polymerase Chain Reaction (RT-PCR) from upper and/or lower respiratory tract specimens. ^9^ However, positive RT-PCR results were not only found from respiratory tract specimen but also from gastrointestinal tract specimens like feces (29% – 53.42% positivity rate), anal swab, and endoscopic biopsy specimens from the stomach, duodenum, ileum, and rectum. ^10^^–^^13^ The RT-PCR test using an anal swab has a good specificity for confirming COVID-19 cases, which is 93.8%. ^12^ The role of anal swab sampling during the COVID-19 pandemic is getting more attention from researchers for several reasons. Severe COVID-19 patients showed higher detectable levels of viral RNA, high viral load, and early positive detection in anal swabs that can affect disease progression. ^14^ Other studies around the world have shown different results and similar studies have not been found in Indonesia. ^5^^,^^6^ This study aims to determine the relationship between anal swab PCR for SARS-CoV-2 with gastrointestinal clinical manifestations and severity in COVID-19 patients in Indonesia.

## Methods

### Study design

This study is a branch of the main research entitled “The Value of Anal Swab RT-PCR for Diagnosis of COVID-19 in Adults in Indonesia”. ^15^ This was an analytic study with a cross-sectional design. This research data in this manuscript is taken from the main research data. There were three potential source of bias, i.e. recruitment bias, allocation bias, and measurement bias. To anticipate the recruitment bias, the author was choosing a sampling method from the main research, which was taken with consecutive sampling. To anticipate the allocation bias, the author was examining anal PCR SARS-CoV-2 swabs at the Virology and Cancer Pathobiology Research Center RSCM-FKUI, which has been verified by the Ministry of Health of the Republic of Indonesia. To anticipate the measurement bias, the author was examining patients directly to collect the demographic data, clinical manifestations, severity, and anal swab PCR SARS-CoV-2 into the main study. The classification of the severity of COVID-19 infection is carried out according to the classification from the Ministry of Health of the Republic of Indonesia.

This research was an analytic study with a cross-sectional design using an etiological approach. The cross-sectional study design allows for an assessment of risk factors and their impact at the same time. Thus this study design excels in convenience, cost efficiency, and does not require re-evaluation for the patients included.

### Ethical statement

This study was approved by the Research Ethics Committee of Universitas Indonesia, Jakarta (Ethical Approval Number KET-639/UN2.F1/ETIK/PPM.00.02/2020), on June 22 ^nd^ 2020. Data was collected between July 2020 until January 2021. Written informed consent was obtained from all patients to participate in this study and publication of the patients’ details.

### Sample

The sample were adult confirmed COVID-19 patients with a positive nasopharyngeal swab PCR who were hospitalized at dr. Cipto Mangunkusumo (RSCM), Mitra Keluarga Hospital Depok, Mitra Keluarga Kelapa Gading Hospital, and Ciputra Hospital during the period July 2020 to January 2021. This time frame was chosen because of the COVID-19 cases in Indonesia were begin to increase nationally at that time point. Minimal respondents for this study is 96, based on formula for two independent samples. The target population is patients with COVID-19 infection above or equal to 18 years, with gastrointestinal manifestations who are hospitalized at selected hospitals for a specified period of time. This study used total sampling from the main research that were using consecutive sampling. The subjects were selected using consecutive sampling and subject inclusion criteria were patients admitted to a COVID-19 hospital who were confirmed positive for nasopharyngeal or nasal swabs using RT-PCR and aged over 18 years. Subject exclusion criteria were patients in critical condition at the time of initial hospital admission or patients who had hematochezia, melena, or other anatomical/physiological abnormalities that did not allow anal swab sampling.

### Data collection

Patients who met the inclusion criteria were asked to participate in this study by signing the informed consent statement. The data collected included: the patient’s characteristics, clinical manifestations, disease severity, and results of the SARS-CoV-2 PCR anal swab using a case report form (CRF) which comes from both the patients directly through interview and their written medical records.

### Data analysis

The collected data were processed and analyzed using SPSS software, version 25.0. Patient characteristics were reported using simple statistics such as n (number of patients), % (percentage of patients), and mean (SD). The relationship between anal SARS-CoV-2 swab PCR with gastrointestinal clinical manifestations and the severity of COVID-19 infection was assessed with a chi-square categorical comparative statistical test and the prevalence ratio (PR) with a 95% confidence interval. Other factors related to the dependent factor were assessed using multivariate logistic regression analysis and calculated odds ratio (OR) with 95% confidence interval.

## Results

### Patient characteristics

**The flow of patients recruited in this study were shown in this diagram:**


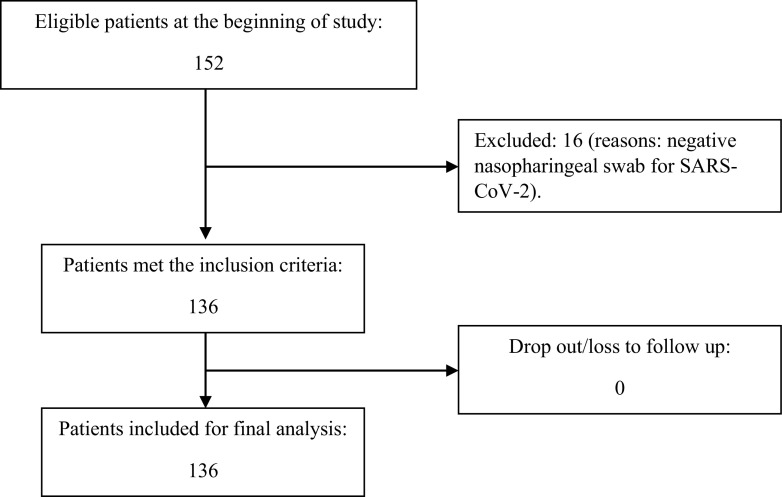


There were 136 patients included in this study, consisting of 68 women (50%) and 68 men (50%) with an average age of 44.1 years **(** Table 1 **)**. ^41^ There were 15 people (11.0%) who were active-smokers. The most common comorbidities found in patients were hypertension 17.6% (n=24), diabetes mellitus 15.4% (n= 21), followed by other lung diseases 6.6% (n=9). There were 52 patients (38.2%) who had positive SARS-CoV-2 anal PCR swabs and 84 patients (61.8%) had negative anal swabs. The most common gastrointestinal clinical manifestations were nausea and vomiting in 69 patients (50.7%), decreased appetite in 62 patients (45.6%), and abdominal pain in 31 patients (22.8%). There were 114 patients (83.8%) classified as mild-moderate and 22 patients (16.2%) classified as severe-critical.

**Table 1.** Patient characteristics.

Variable	Total (n = 136)
Age, mean (SD)	44.1 (12.604)
Gender, n (%)	
Men	68 (50.0)
Women	68 (50.0)
Smoking, n (%)	
Active smoker	15 (11.0)
Non-smokers	121 (89.0)
Comorbidities, n (%)	
Hypertension	24 (17.6)
Diabetes Mellitus	21 (15.4)
Other lung diseases	9 (6.6)
Cardiovascular disease	8 (5.9)
Malignancy	3 (2.2)
Chronic Obstructive Pulmonary Disease	1 (0.7)
Clinical symptoms, n (%)	
Nausea-vomiting	69 (50.7)
Loss of appetite	62 (45.6)
Abdominal pain	31 (22.8)
Diarrhea	17 (12.5)
Constipation	5 (3.7)
Degree of severity at hospital admission, n (%)	
Mild-moderate	114 (83.8)
Severe-critical	22 (16.2)

n = number of patients; % = percentage of patients; smoking status = active smoking status at time of procedure. SD = standard deviation.

### SARS-CoV-2 anal swab PCR results and gastrointestinal clinical manifestations

There was a statistically significant relationship between the anal swab SARS-CoV-2 PCR and gastrointestinal clinical manifestations in the form of diarrhea or nausea and vomiting (P = 0.031, PR = 1.546) shown in Table 2. This was demonstrating that the SARS-CoV-2 anal swab PCR can be used as a sign or risk factor for gastrointestinal clinical manifestations in the form of diarrhea or nausea and vomiting.

**Table 2.** Results of chi-square PCR SARS-CoV-2 anal swab with gastrointestinal clinical manifestations.

		Gastrointestinal clinical manifestation			P Value	PR
		No	Yes	Total		
Anal Swab PCR for SARS-CoV-2	Negative	45	39	84	0.031	1.546
	Positive	18	34	52		
	Total	63	73	136		

P value calculated using chi square test.

PR = Prevalence ratio; PCR = polymerase chain reaction.

Factors that influence gastrointestinal clinical manifestation in COVID-19 patients were assessed with a multivariate logistic regression analysis test. The factors were anal swab, comorbidities, smoking, male gender, and age over 50 years old. The first stage was the selection of candidate variables that will enter the multivariate logistic regression model ( Table 3). The variables anal swab, male gender, and age over 50 years old have P-value <0.25, so these variables deserved to be included in the multivariate model.

**Table 3.** Selection of candidate variables for logistics multivariate regression model.

Variable	P Value
Anal Swab	0.033
Comorbidity	0.606
Smoking	0.977
Male gender	0.123
>50 years old	0.215

Significance if P value < 0.25.

The results of statistical tests of multivariate logistic regression analysis to assess the factors that influence gastrointestinal clinical manifestations are described in Table 4. The anal swab has the greatest statically relationship with the severity of COVID-19 infection (P = 0.035) and an adjusted OR value of 2.184 (95% CI = 1.054-4.525). Another male gender variable has a P-value of 0.108 and an adjusted OR of 0.564 (95% CI = 0.281-1.134). The variable age over 50 years old has a P-value of 0.318 and an adjusted OR of 1.467 (95% CI = 0.691-3.112). Thus, the anal swab variable was an independent variable that affects the clinical manifestations of the gastrointestinal tract. In patients with the same gender and age, patients with positive anal swab were twice more likely to experience gastrointestinal clinical manifestations of diarrhea or nausea and vomiting, compared with negative anal swabs patients.

**Table 4.** Multivariate logistic regression factors influencing gastrointestinal clinical manifestations.

Variable	Adjusted OR	95% CI	P Value
Anal Swab	2.184	1.054-4.525	0.035
Male gender	0.564	0.281-1.134	0.108
>50 years old	1.467	0.691-3.112	0.318

P value calculated using multivariate logistic regression analysis.

OR = Odds Ratio; CI = Confidence Interval.

### SARS-CoV-2 anal swab PCR results and severity of COVID-19 infection

There was no statistically significant relationship between the SARS-CoV-2 anal swab PCR and the severity of COVID-19 infection (P = 0.844, PR = 0.984) shown in Table 5. This was demonstrating that the SARS-CoV-2 anal swab PCR cannot be used as a sign or risk factor for the severity of COVID-19 infection.

**Table 5.** Results of chi-square PCR SARS-CoV-2 anal swab with the severity of COVID-19 infection.

		Severity classification			P Value	PR
		Mild-moderate	Severe-critical	Total		
Anal Swab PCR for SARS-CoV-2	Negative	70	14	84	0.844	0.984
	Positive	44	8	52		
	Total	114	22	136		

P value calculated using chi square test.

PR = Prevalence Ratio; PCR = Polymerase Chain Reaction.

Factors that influence the severity of COVID-19 infection were assessed with a multivariate logistic regression analysis test. The factors were anal swab, comorbidities, smoking, male gender, and age over 50 years old. The first stage was the selection of candidate variables that will enter the multivariate logistic regression model ( Table 6). The variables “Comorbid” and “Smoking” had P-value <0.25, so these variables deserved to be included in the multivariate model. The variable “Anal swab” did not qualify for inclusion in the multivariate model because it had a P-value > 0.25, but was still included to assess the relationship of the anal swab in the multivariate model.

**Table 6.** Selection of candidate variables for logistics multivariate regression model.

Variable	P Value
Anal Swab	0.844
Comorbidity	0.154
Smoking	0.002
Male gender	0.642
>50 years old	0.661

Significance if P value < 0.25.

The results of statistical tests of multivariate logistic regression analysis to assess the factors that influence the severity of COVID-19 infection are described in Table 7. Smoking has the greatest statically relationship with the severity of COVID-19 infection (P = 0.003) and an adjusted OR value of 5.920 (95% = 1.839-19.061). The comorbid variable which has a P-value of 0.222 and adjusted OR 1.906 (95% CI = 0.676-5.372). The anal swab variable has a P-value of 0.940 and an adjusted OR of 0.962 (95% CI = 0.350-2.643). Thus, it demonstrates that the “Smoking” variable is an independent variable that affects the severity of COVID-19 infection. In patients with the same comorbidities and anal swab results, patients who smoked were almost 6 times more likely to have a more severe COVID-19 infection than patients who did not smoke.

**Table 7.** Logistics multivariate regression factors influencing the severity of COVID-19 infection.

Variable	Adjusted OR	95% CI	P Value
Smoking	5.920	1.839-19.061	0.003
Comorbidity	1.906	0.676-5.372	0.222
Anal Swab	0.962	0.350-2.643	0.940

P value calculated using multivariate logistic regression analysis.

OR = Odds Ratio; CI = Confidence Interval.

## Discussion

Various kinds of research around the world continue to be carried out about SARS-CoV-2 infection and the involvement of the gastrointestinal tract with the resulting clinical manifestations. The primary outcome of this study was to determine the relationship between anal swab PCR for SARS-CoV-2 with gastrointestinal clinical manifestations and the severity in COVID-19.

In this study, only 38.2% of the patients showed positive anal swab results to detect SARS-CoV-2 infection. A different proportion was found in the research by Gong-Qi *et al*. being as many as 53.6% of patients showed a positive result of SARS-CoV-2 anal swab. ^16^ The most common gastrointestinal manifestations in this study were nausea and vomiting (50.7%), decreased appetite (45.6%), and abdominal pain (22.8%). In the systematic review and meta-analysis of Ren Mao *et al*., the most common gastrointestinal manifestations were decreased appetite (21%), diarrhea (9%), and nausea and vomiting (6%). ^17^ The severity of COVID-19 in the study by Weiyin Lin *et al*. was 81.2% classified as mild-moderate and 19.8% as severe-critical. ^7^

In this study, there was a statistically significant relationship between the SARS-CoV-2 anal swab PCR variable and gastrointestinal clinical manifestations in the form of diarrhea or nausea and vomiting (P = 0.031). Similar findings were found in the study by Chaoqun Han *et al*. that concluded there was a statistically significant relationship between the detection of SARS-CoV-2 genetic material in feces and clinical manifestation of diarrhea or nausea and vomiting in patients (P = 0.033). ^18^ The opposite results were obtained in the study of Yong Zhang *et al*. The analysis results showed that the P-value was 0.0503, which means that there is no statistically significant relationship between the detection of SARS-CoV-2 genetic material in feces and clinical manifestation of diarrhea in patients. ^19^

At the time of writing this study, there is no definite reason or theory to explain the relationship between PCR SARS-CoV-2 anal swab and gastrointestinal clinical manifestations. Several hypotheses to support the relationship suggest a direct infection of SARS-CoV-2 to the gastrointestinal tract, direct invasion of the virus can damage the existing protective function of the gastrointestinal tract and increase permeability. ^18^ This will facilitate the invasion of pathogens in the gastrointestinal tract and cause clinical manifestations in the form of diarrhea, malabsorption of nutrients, and others. ^18^

In this study, it was found that there was no statistically significant relationship between the SARS-CoV-2 anal swab PCR and the degree of COVID-19 severity. Similar findings were obtained in the study of Weiyin Lin *et al*. that showed there was no statistically significant relationship between the SARS-CoV-2 anal swab PCR variable and the severity of both during hospital admission (P = 0.097) and during treatment (P = 0.229). ^7^ The opposite results were obtained in the Gong-Qi study. *et al*. that there was a statistically significant relationship between the positive anal swab results for SARS-CoV-2 RNA and the degree of severity (P = 0.029). ^16^

Several studies showed results that were contrary to this study. The reasons underlying why other studies have concluded that there is a significant relationship between PCR SARS-CoV-2 anal swabs and the severity of COVID-19 infection are still largely unknown. Several hypotheses suggest that the high viral replication in the respiratory system allows the virus to actively penetrate the alveolus and endothelium of blood vessels to enter the bloodstream and infect the gastrointestinal epithelium. ^7^ Another hypothesis is that SARS-CoV-2 RNA is found in various locations of the gastrointestinal tract, starting from the esophagus, stomach, duodenum, and rectum. The wide availability of ACE2 receptors in the gastrointestinal tract can be a site of viral replication outside the lungs. Thus, the SARS-CoV-2 elimination from the respiratory system would be delayed, which can cause disease progression. ^7^ The different results between this study and other studies could also be due to the limited number of samples and the unbalanced proportion of samples classified as mild-moderate and severe-critical.

Several factors were analyzed to assess their effect on gastrointestinal clinical manifestations, were anal swab, comorbidities (hypertension, diabetes mellitus, chronic obstructive pulmonary disease [COPD]), smoking, male gender, and age over 50 years old. Data analysis showed that the variables “Anal swab”, “Male gender”, and “Age over 50 years” had an influence on gastrointestinal clinical manifestations with “Anal swab” being the most dominant factor in influencing gastrointestinal clinical manifestations. ACE2 receptors in the gastrointestinal tract are the entry point for viruses to enter host cells. ^6^^,^^8^^,^^11^^,^^20^ Viruses can replicate and spread to various locations in the body. Viral infections will damage the intestinal mucosa and cause gastrointestinal manifestations in the form of diarrhea. ^21^ In certain individuals, a cytokine storm may occur due to the infection that will exacerbate the occurrence of this diarrhea. The body's response to the cytokine storm that occurs will cause intestinal hypoxia to tissue ischemia which also contributes to diarrhea. The existing inflammation affects the balance of microorganisms in the gastrointestinal tract which will lead to more prominent gastrointestinal symptoms. ^20^^,^^22^

Comorbid factors did not affect gastrointestinal clinical manifestations in this study. The opposite finding was found in the study by Uday C. *et al*. The study performed a multivariate analysis to assess factors associated with gastrointestinal manifestations. The analysis results showed that patients with comorbidities had an adjusted OR of 6.87 (95% CI = 1.22–38.79) and a P value of 0.027. ^23^

In this study, smoking did not affect gastrointestinal clinical manifestations. This finding was supported by the research of Ting Zhan *et al*. that showed there is no statistically significant relationship between smoking history and gastrointestinal manifestations (P = 0.501). ^24^

Male gender had a correlation with gastrointestinal clinical manifestations in this study; men were more likely to be infected by bacteria and viruses than females. This may be because women have strong innate and adaptive immune responses. ^25^ However, an opposing study showed that gastrointestinal symptoms in the form of decreased appetite (p-value <0.001) and diarrhea (p-value 0.002) in COVID-19 infection were significantly more common in women compared to men. ^26^

The factor “Age over 50 years old” has an influence on gastrointestinal clinical manifestations in this study which is supported by other studies. A similar study with multivariate analysis showed gastrointestinal symptoms including diarrhea, nausea, vomiting, abdominal pain, and decreased appetite had a positive association with the patient’s age. Human aging is associated with innate and adaptive immunity decline, and will gradually lose the defense function to against infection. Thus, older adults are more vulnerable to COVID-19 infection. ^27^

In this study, several factors were assessed for their influence on the severity of COVID-19 infection. Those factors were anal swab, comorbidities (hypertension, diabetes mellitus, COPD), smoking, male gender, and age over 50 years old. Data analysis showed that the variables “Comorbid” and “Smoking” had an influence on the severity of COVID-19 infection with “Smoking” being the most dominant factor. A systematic review by J.E. Rod *et al*. processed data from 17 studies and collected up to 60 predictors or factors that influence the severity of COVID-19 infection. Factors tested using multivariate analysis, i.e. age, C-reactive protein, D-dimer, albumin, body temperature, Sequential Organ Failure Assessment (SOFA) score, and diabetes. The results of the analysis showed that diabetes is the most consistent comorbidity in predicting the severity of COVID-19 infection. Patients with diabetes mellitus may experience an over-inflammatory response and a hypercoagulable state that is associated with dysregulation of glucose metabolism. Both have an effect on the aggravation of the disease. ^28^ Patients with comorbid hypertension are known to be at higher risk for respiratory tract infections and an increased incidence of ICU admissions. ^29^^,^^30^ In patients with COPD, there has been the destruction of the lung parenchyma, chronic inflammation, airflow limitation, and the presence of infection could trigger COPD exacerbations. Thus, patients with COPD have a worse prognosis and more severe disease. Smoking behavior factors are known to cause disorders of the immune system in the lungs, prolonged lung structure damage, and increased ACE2 expression which underlies the degree of disease that occurs. ^31^^–^^34^ Thus, smoking patients can influence the COVID-19 severity.

In this study, the male gender was not included in the multivariate regression analysis because it did not have a statically significant influence on the severity of COVID-19. Several studies showed the relationship between gender and COVID-19 infection. Male gender is related to differences in behavior, social activities, roles in society, smoking and alcohol consumption. Comorbidities like hypertension, cardiovascular disease, and COPD is more likely in men which can influence the prognosis of COVID-19. In men, ACE2 expression is higher and the cytokine storms are increased compared to women. ^35^^–^^37^

The age over 50 years old variable did not affect the severity of COVID-19, thus it was not included in the multivariate regression analysis model in this study. Several studies had opposite results that showed aging is associated with an impaired mucociliary clearance which facilitates infection, weaker innate and adaptive immune systems, and coagulation abnormalities which can increase the risk of disseminated intravascular coagulation. Thus, leading to a more severe disease. ^38^^–^^40^

As far as our knowledge, this article is the first cross sectional study that has assessed the relationship between SARS-COV-2 anal swab PCR with gastrointestinal clinical manifestations and the severity of COVID-19 in Indonesia this far. Beside it has several limitations, i.e. the nature design of cross sectional study that cannot establish the long term causal effect of SARS-CoV 2 infection and the gastrointestinal symptoms, nor measuring the long term gastrointestinal outcome of this disease. Also, the SARS CoV-2 PCR only evaluate the presence of genetic materials in the specimen collected, but cannot establish the viability of the virus itself.

## Conclusion

This study demonstrated that there is a statically significant relationship between PCR SARS-CoV-2 anal swab with gastrointestinal clinical manifestations (diarrhea or nausea and vomiting). Therefore, SARS-CoV-2 anal swab PCR can be used as a sign or risk factor for gastrointestinal clinical manifestations. There is no statistically significant relationship between the SARS-CoV-2 anal swab PCR and the severity of COVID-19 infection. Therefore, SARS-CoV-2 anal swab PCR cannot be used as a sign or risk factor for the severity of COVID-19 infection. Patients with gastrointestinal manifestations such as diarrhea or nausea and vomiting need to be more aware of the potential for COVID-19 infection, even though they do not experience respiratory manifestations. Patients with suspected COVID-19 infection with gastrointestinal manifestations (diarrhea, nausea, and vomiting) who show negative nasopharyngeal swab results are advised to have an anal swab examination. In this study, the number of samples analyzed had met the minimum requirement for a representative analysis, but analysis using a larger number of samples was needed to further assess the relationship between the anal swab SARS-CoV-2 PCR test results with gastrointestinal clinical manifestations and COVID-19 severity. The outcomes of the study can be considered to increase awareness of the possibility of fecal-oral transmission.

## Data Availability

Open Science Framework: Relationship between Anal Swab PCR for SARS-CoV-2 with Gastrointestinal Clinical Manifestations and Severity of COVID-19 Infection in Indonesia, https://doi.org/10.17605/OSF.IO/9E24G. ^41^ This project contains the following underlying data:
- Raw data.xlsx (this file contains raw questionnaire and anal swab results) Data are available under the terms of the Creative Commons Attribution 4.0 International license (CC-BY 4.0).
