# Supplementary material for: Relationship between anal swab PCR for SARS-CoV-2 with gastrointestinal clinical manifestations and severity of COVID-19 infection in Indonesia
Source: F1000Res. 2023 Aug 7;12:358. Originally published 2023 Mar 31. [Version 2] doi: 10.12688/f1000research.128821.2 (PMC10521035; doi:10.12688/f1000research.128821.2)
Supplement: Peer review history

# Reviewer report: Reviewer response for version 2

**DOI:** 10.5256/f1000research.154185.r194526

**Published:** 2023-09-06

**Authors:** Fauzi Yusuf

**Affiliations:** 1 Division of Gastroenterology-Hepatology, Department of Internal Medicine, Faculty of Medicine, Universitas Syiah Kuala, Banda Aceh, Aceh, Indonesia

Gastrointestinal symptoms might develop in COVID-19 patients. Virus RNA can be detected in fecal samples even after respiratory samples already turned test negative. Numerous research state that SARS-CoV-2 virus infection, which causes COVID-19, gives rise to both respiratory and gastrointestinal symptoms and confirms that RNA virus has been detected in the patient’s fecal sample.

 This research discusses that initial presentation of enteric manifestations of SAR-CoV-2 as an interesting diagnostic challenge to clinicians in diagnosing patients with COVID-19. It also indicates potential fecal transmission of this virus. The content of the abstract is already consistent, structured, and informative.

 The research’s background, problem formulation, objectives, literature review and novelty are connected and relevant.

 The content of the study has consistent material, well-structured theoretical framework, authentic data, and precise information supported by informative tables and figures.

 In the discussion part, all relevant and important points are well-discussed.

 By rising number of COVID-19 reported cases, there is an urgent need for further research on the enteric manifestations of COVID-19 and the temporal pattern of the SARS-CoV-2 virus in fecal excretion. This study will support the diagnosis made by particularly gastroenterologists and endoscopists who may not be familiar with this disease.

 Although the study design has potential bias, this analytic study with cross-sectional design is sufficient to allow study repetition by others.

 I think the conclusions already support the results but further research about possible fecal oral transmission should be recommended.

Is the work clearly and accurately presented and does it cite the current literature?

Yes

If applicable, is the statistical analysis and its interpretation appropriate?

Yes

Are all the source data underlying the results available to ensure full reproducibility?

Yes

Is the study design appropriate and is the work technically sound?

Yes

Are the conclusions drawn adequately supported by the results?

Yes

Are sufficient details of methods and analysis provided to allow replication by others?

Yes

Reviewer Expertise:

Gastroenterology, Hepatology, Microbiota, Short Chain Fatty Acid, Helicobacter pylory, Covid 19, Colorectal cancer

I confirm that I have read this submission and believe that I have an appropriate level of expertise to confirm that it is of an acceptable scientific standard.

# Reviewer report: Reviewer response for version 2

**DOI:** 10.5256/f1000research.154185.r194527

**Published:** 2023-09-05

**Authors:** Hery Djagat Purnomo

**Affiliations:** 1 Faculty of Medicine, Division of Gastroenterohepatology, Department of Internal Medicine, dr Kariadi Hospital, Universitas Diponegoro, Semarang, Central Java, Indonesia

This revised article was appropriate and response our previous comment and suggestion. It's enough and no further comments, please for indexing. (Approved).

Is the work clearly and accurately presented and does it cite the current literature?

Yes

If applicable, is the statistical analysis and its interpretation appropriate?

Yes

Are all the source data underlying the results available to ensure full reproducibility?

Yes

Is the study design appropriate and is the work technically sound?

Partly

Are the conclusions drawn adequately supported by the results?

Partly

Are sufficient details of methods and analysis provided to allow replication by others?

Yes

Reviewer Expertise:

Gastroenterology (Colorectal cancer screening)  Hepatology (NAFLD, Hepatitis B, C, Hepatocellular Carcinoma)

I confirm that I have read this submission and believe that I have an appropriate level of expertise to confirm that it is of an acceptable scientific standard.

# Reviewer report: Reviewer response for version 1

**DOI:** 10.5256/f1000research.141446.r172704

**Published:** 2023-07-31

**Authors:** Hery Djagat Purnomo

**Affiliations:** 1 Faculty of Medicine, Division of Gastroenterohepatology, Department of Internal Medicine, dr Kariadi Hospital, Universitas Diponegoro, Semarang, Central Java, Indonesia

This is a cross-sectional study investigating the association between anal swab PCR for SARS-CoV-2 with gastrointestinal clinical manifestations and severity of COVID-19 infection. This study has many strengths including being a clear manuscript, uses some of new available datasets for analysis, and careful interpretation of the findings. However, some concerns remain as detailed below :

1. In the study design, research biases are more suitable to be included in the study limitations. There is repetition of the same sentence “this research was an analytic study with a cross-sectional design using an etiological approach”.
  Furthermore, the advantage of the study design that there is no need for re-evaluation should not be mentioned, because to determine the association between anal swab PCR with disease severity, it would be better if it’s done periodically/longitudinal.
2. The Inclusion and exclusion criteria did not mention the history of Gastrointestinal disease. Was there any influence to the GI symptoms of the subjects?
3. Regarding comorbidities evaluation, the comorbidities were evaluated individually or as in a group of yes or no comorbidity?
4. In the results explanation of table 4.; it was written “The anal swab has the greatest statically relationship with the severity of COVID-19 infection (P = 0.035) and an adjusted OR value of 2.184 (95% CI = 1.054-4.525)”, while Table 4 is the result of multivariate regression factors that contribute to GI manifestations. Could you please correct?
5. In the sentence “Another male gender variable has a P-value of 0.108 and an adjusted OR of 0.564 (95% CI = 0.281-1.134)” ; it would be better using “moreover/furthermore/additionally”.
6. In the discussion, it has not been well explained the reason of the comorbidity analysis which is different from previous studies.
7. Given the limitation of the study, it is more appropriate to say that there is not enough evidence to support the association between Anal swab PCR and disease severity. It is due to study design that the evaluation was done once in the admission. Furthermore, there are others factors such as drugs, other comorbidities, virus viability that may influence the results.
8. Please consider adding suggestions for future research.
9. The conclusions are too long and inconclusive. Please consider to write in an concise sentence and show the impact of this research on clinical applications. Then “analysis using a larger number of samples was needed” should not be included in the conclusion.

Is the work clearly and accurately presented and does it cite the current literature?

Yes

If applicable, is the statistical analysis and its interpretation appropriate?

Yes

Are all the source data underlying the results available to ensure full reproducibility?

Yes

Is the study design appropriate and is the work technically sound?

Partly

Are the conclusions drawn adequately supported by the results?

Partly

Are sufficient details of methods and analysis provided to allow replication by others?

Yes

Reviewer Expertise:

Gastroentero Hepatologist, Internist

I confirm that I have read this submission and believe that I have an appropriate level of expertise to confirm that it is of an acceptable scientific standard, however I have significant reservations, as outlined above.

# Response

**Published:** 2023-08-04

**Authors:** Murdani Abdullah

**Affiliations:** University of Indonesia, Indonesia

**1. In the study design, research biases are more suitable to be included in the study limitations. There is repetition of the same sentence “this research was an analytic study with a cross-sectional design using an etiological approach”.** **Furthermore, the advantage of the study design that there is no need for re-evaluation should not be mentioned, because to determine the association between anal swab PCR with disease severity, it would be better if it’s done periodically/longitudinal.**

 Thank you for your input. We have moved the research biases in the study design to our study limitation. We also have deleted the same sentence in the article and not mentioned the need for re-evaluation.

 2. **The Inclusion and exclusion criteria did not mention the history of Gastrointestinal disease. Was there any influence to the GI symptoms of the subjects?**

 Thank you for your input. We did not mention the history of gastrointestinal disease in the inclusion and exclusion criteria because there was no influence of the history of gastrointestinal disease with the gastrointestinal symptoms of the subjects. The gastrointestinal symptoms that are assessed in this study are the symptoms that appear in the present.

 3. **Regarding comorbidities evaluation, the comorbidities were evaluated individually or as in a group of yes or no comorbidity?**  

 Thank you for your input. The comorbidities were evaluated in a group of yes or no comorbidity

**4. In the results explanation of table 4.; it was written “The anal swab has the greatest statically relationship with the severity of COVID-19 infection (P = 0.035) and an adjusted OR value of 2.184 (95% CI = 1.054-4.525)”** **, while Table 4 is the result of multivariate regression factors that contribute to GI manifestations. Could you please correct?**

 Thank you for your input. We have already corrected the sentence into “The anal swab has the greatest statistically relationship that influence gastrointestinal clinical manifestations (P=0.035) with an adjusted OR value of 2.184 (95% CI = 1.054-4.525)”.

**5. In the sentence “Another male gender variable has a P-value of 0.108 and an adjusted OR of 0.564 (95% CI = 0.281-1.134)” ; it would be better using “moreover/furthermore/additionally”.**

 Thank you for your input. We have changed the word “another” into “additionally”.

**6. In the discussion, it has not been well explained the reason of the comorbidity analysis which is different from previous studies.**

 Thank you for your input. We have added the reason of the comorbidity analysis which is different from previous studies in the article.

**7. Given the limitation of the study, it is more appropriate to say that there is not enough evidence to support the association between Anal swab PCR and disease severity. It is due to study design that the evaluation was done once in the admission. Furthermore, there are others factors such as drugs, other comorbidities, virus viability that may influence the results.**

 Thank you for your input. We have combined the sentence you suggest into the article. 

**8. Please consider adding suggestions for future research.**

 Thank you for your input. We have added the suggestions for future research in our article.

**9. The conclusions are too long and inconclusive. Please consider to write in an concise sentence and show the impact of this research on clinical applications. Then “analysis using a larger number of samples was needed” should not be included in the conclusion.**

 Thank you for your input. We have shortened the conclusion and the sentence “analysis using a larger number of samples was needed” has been deleted in the conclusion.

# Reviewer report: Reviewer response for version 1

**DOI:** 10.5256/f1000research.141446.r168691

**Published:** 2023-06-19

**Authors:** Fauzi Yusuf

**Affiliations:** 1 Division of Gastroenterology-Hepatology, Department of Internal Medicine, Faculty of Medicine, Universitas Syiah Kuala, Banda Aceh, Aceh, Indonesia

Gastrointestinal symptoms occur in patients with COVID-19. Virus RNA can be detected in fecal samples even those collected after respiratory samples test negative.

 There are many reports that Infection with SARS-CoV-2 virus, which causes COVID-19, results in respiratory as well as gastrointestinal symptoms and virus RNA has been detected in fecal samples.

 This manuscript addresses enteric manifestations of SAR-CoV-2 not only as an interesting diagnostic challenge to clinicians when facing patients with COVID-19 symptoms on initial presentation but also signifies potential fecal transmission of this virus.

 The content of the abstract is already appropriate, structured, and informative.

 The research’s background, problem formulation, objectives, literature review and novelty are relevant.

 The content of the paper has consistent material, good theoretical framework, valid data, and appropriate information supported by clear tables and figures.

 In the discussion part, all relevant matters are well-discussed.

 With the increasing number of reported cases of COVID-19, there is a pressing need for more research on the enteric manifestations of COVID-19 and the temporal pattern of fecal shedding of the SARS-CoV-2 virus, particularly to gastroenterologists and endoscopists who may not be familiar with this disease.

 Even though the study design has potential bias, this analytic study with cross-sectional design is sufficient to allow application by others.

 I think the conclusions drawn adequately support the results, but still need more research about possible fecal oral transmission.

Is the work clearly and accurately presented and does it cite the current literature?

Yes

If applicable, is the statistical analysis and its interpretation appropriate?

Yes

Are all the source data underlying the results available to ensure full reproducibility?

Yes

Is the study design appropriate and is the work technically sound?

Yes

Are the conclusions drawn adequately supported by the results?

Yes

Are sufficient details of methods and analysis provided to allow replication by others?

Yes

Reviewer Expertise:

Gastroenterology, Hepatology, Microbiota, Short Chain Fatty Acid, Helicobacter pylory, Covid 19, Colorectal cancer

I confirm that I have read this submission and believe that I have an appropriate level of expertise to confirm that it is of an acceptable scientific standard.
